# Genome-Wide Identification, Classification, and Expression Profiling Reveals R2R3-MYB Transcription Factors Related to Monoterpenoid Biosynthesis in *Osmanthus fragrans*

**DOI:** 10.3390/genes11040353

**Published:** 2020-03-26

**Authors:** Hai-Yan Li, Yuan-Zheng Yue, Wen-Jie Ding, Gong-Wei Chen, Ling Li, Yu-Li Li, Ting-Ting Shi, Xiu-Lian Yang, Liang-Gui Wang

**Affiliations:** 1 Key Laboratory of Landscape Architecture, Jiangsu Province, College of Landscape Architecture, Nanjing Forestry University, Nanjing 210037, China; tarashiyumi@163.com (H.-Y.L.); yueyuanzheng@njfu.edu.cn (Y.-Z.Y.); wenjieding@njfu.edu.cn (W.-J.D.); chengongwei0118@163.com (G.-W.C.); LLDREAM0909@163.com (L.L.); chestnutlyl@163.com (Y.-L.L.); shitingting313@163.com (T.-T.S.); yangxl339@126.com (X.-L.Y.); 2 Co-Innovation Center for Sustainable Forestry in Southern China, Nanjing Forestry University, Nanjing 210037, China

**Keywords:** *Osmanthus fragrans*, 2R-MYB transcription factor, gene evolution, phylogenetic analysis, monoterpenoid biosynthesis, linalool oxides

## Abstract

*Osmanthus fragrans* is widely grown for the purpose of urban greening and the pleasant aroma emitted from its flowers. The floral scent is determined by several monoterpenoid volatiles, such as linalool and its oxides, which are a few of the most common volatiles and the main components of the essential oils in most sweet osmanthus cultivars. In addition, the relative contents of *cis*- and *trans*-linalool oxide (furan) may affect the aromas and quality of the essential oils. MYB proteins represent the largest family of transcription factors in plants and participate in regulating secondary metabolites. Several *cis*-elements, especially AC-rich regions, are known to be bound by 2R-MYBs and could be found in the promoter of the enzyme genes in the terpenoid metabolic pathway. However, there has to date been no investigation into the 2R-MYB family genes involved in regulating terpenoid biosynthesis in *O. fragrans*. Here, 243 non-redundant 2R-MYB proteins were grouped into 33 clusters based on the phylogeny and exon-intron distribution. These genes were unevenly distributed on 23 chromosomes. Ka/Ks analysis showed that the major mode of 2R-MYB gene evolution was purifying selection. Expression analysis indicated that 2R-MYB genes in *O. fragrans* exhibited varied expression patterns. A total of 35 OfMYBs representing the highest per kilobase per million mapped reads in the flower were selected for quantitative real-time PCR analysis. The correlation analysis between the expression level and the contents of fragrant compounds at different flowering stages suggested that *OfMYB19*/*20* exhibited remarkably positive correlation with the accumulation of *cis*-linalool oxides. *OfMYB51*/*65*/*88*/*121*/*137*/*144* showed significantly negative correlations with one or more linalool oxides. Characterization of these proteins revealed that OfMYB19 and OfMYB137 were localized in the nuclei, but did not show transcriptional activation in the yeast system, which suggested that they may be bound to other transcription factors to exert regulatory functions. These findings provide useful information for further functional investigation of the 2R-MYBs and offer a foundation for clarifying the 2R-MYB transcription factors involved in the molecular mechanism of the regulation of monoterpenoid biosynthesis in *Osmanthus fragrans*.

## 1. Introduction

Transcription factors (TFs) are important proteins that bind special *cis*-acting elements to affect the expression of target genes by modulating the transcription rate [1]. TFs can be categorized into distinct families in accordance with their DNA-binding domains [2]. MYB TFs form a very large family of proteins and are highly conserved among animal, plant, and yeast homologs, which all have an extremely conserved MYB domain composed of 1~4 adjacent repeats (R) and containing about 52 amino acids [3,4]. According to the number of adjacent repeats, MYB TFs include 1R (R1/2, R3-MYB), 2R (R2R3-MYB), 3R (R1R2R3-MYB), and 4R [3,5]. In contrast to 3R-MYB proteins, which are generally found in animals in limited amounts, plants contain a large quantity of 2R-MYB proteins. As increasing numbers of plants have undergone genome sequencing, many 2R-MYB TFs have been characterized. For example, 126 members of the 2R-MYB gene family were reported in *Arabidopsis thaliana* [3], 244 in soybean [6], 185 in pear [7], 122 in tomato [8], and 114 in moso bamboo [9]. A conserved N-terminus and variable domain in the C-terminus ensure that MYB genes play comprehensive regulatory roles in plant growth and development [5,10].

The COLORED1 (C1) locus encoding an MYB domain protein was first identified in plants. This locus is related to anthocyanin synthesis in maize (Zea mays) kernels [11]. Since then, the functions of a considerable number of 2R-MYB proteins have been investigated in different plant species. These proteins are thought to be involved in defense and responses to various biotic and abiotic stresses, cell development, control of the cell cycle, and regulating primary and secondary metabolism [3,5]. In recent years, research has focused more on the regulation of secondary metabolism.

*Osmanthus fragrans*, also named sweet osmanthus, is an evergreen woody flowering plant of the family Oleaceae and known for its fragrant flowers and flavor. The species is also a source of essential oils and is widely cultivated in Asia [12]. The fragrant flowers are used in the food industry to produce tea, foods, and beverages with pleasing aromas. The essential oil that is derived from these plants is a major component of perfumes and fragrances [13]. The largest number of fragrant compounds are released during the full flowering stages of the life cycle. The main floral volatile compounds released from sweet osmanthus are terpenoids, especially monoterpenes including *β*-ionone, *cis*-linalool oxide (furan), *trans*-linalool oxide (furan), linalool, and trans-*β*-ocimene [14,15].

The essential oil is of important economic value, but is found only in low concentrations in *O. fragrans*. The main components of the essential oil are formed through the terpene synthesis pathway, and therefore, investigations into the TF-regulated expression of multiple key genes in the terpenoid metabolic pathway is of interest as a means to increase its production. To date, TFs that have been reported to be involved in the regulation of terpenoids are AP2, bHLH, WRKY, and bZIP in Artemisia, *Salvia miltiorrhiza*, and *Catharanthus roseus* [16,17,18,19,20,21]. There are only a few 2R-MYB transcription factors that have been annotated and elaborated in the terpene metabolic pathways. It was reported that *SmMYB36* and *SmMYB9b* in salvia could positively regulate the biosynthesis of tanshinone [22,23]. Only *MsMYB* has been investigated to be a negative regulator of monoterpene biosynthesis by suppressing the expression of geranyl diphosphate synthase [24]. The roles of 2R-MYB genes and TFs in the terpenoid pathways of *O. fragrans* remain to be elucidated.

In this study, 243 2R-MYB genes of sweet osmanthus were identified and analyzed at the genome-wide level. By comparing data from known *Arabidopsis* MYB genes, systematic genome-wide identification and detailed evolutionary analysis of 2R-MYB genes were performed. The function of each subgroup of the OfMYB family of proteins was predicted by comparative analysis with the *Arabidopsis* MYB family. In addition, quantitative real-time (qRT)-PCR was used, and the transcriptome data of *OfMYB* genes in various organs and flowering stages contributed to revealing the regulatory mechanisms at different developmental stages. According to the correlation analysis between the expression levels and scent compounds during different flowering stages, candidate genes were selected for cDNA isolation, subcellular localization, and transactivation assays. These studies provided useful information for further investigation into the functions of OfMYB proteins in sweet osmanthus.

## 2. Materials and Methods

### 2.1. Identification of OfMYB Genes

A total of 126 2R-MYB protein sequences of *Arabidopsis thaliana* were downloaded from TAIR (http://www.arabidopsis.org/). There was an attempt to obtain putative MYB genes from the sweet osmanthus genome database using the MYB DBD domain (Accession No. PF00249, http://pfam.xfam.org/). All hits with a cutoff value less than 0.01 were collected. Subsequently, the number of domains present in the sequences were determined using the NCBI Batch Web CD-Search Tool and SMART 7.0 software [25]. Finally, 243 putative 2R-MYB genes were identified from sweet osmanthus. The physicochemical characteristics of the MYB protein, such as the lengths of protein sequences, molecular weight (MW), and theoretical isoelectric point (pI), were analyzed with the online tool ExPASy.

### 2.2. Sequence Analysis and Phylogenetic Tree Construction

To obtain the features of the 2R-MYB domain of each sequence, alignment of OfMYB proteins was implemented using the DNAMAN 6.0 tool with default parameters (http://www.lynnon.com). The structures of the *OfMYB* genes were visualized using GSDS 2.0 (http://gsds.cbi.pku.edu.cn/) [26]. The conserved protein motif of each putative OfMYB family member was analyzed by the MEME program (http://meme-suite.org/), with the motif number to be identified set at 20 and the other parameters as default. 2R-MYB protein sequences of sweet osmanthus and *A. thaliana* were aligned using MUSCLE [27]. The phylogenetic analysis was conducted in the MEGA 6.0 program using the Neighbor-Joining (N-J) method with a bootstrap analysis of 1000 replicates, the pairwise deletion of gaps, and a Poisson model [28]. Finally, the phylogenetic tree was decorated in FigTree (v1.4.2) software.

### 2.3. Chromosome Location, Gene Duplication, and Syntenic Analysis

The position information of putative 2R-MYB genes was extracted from the General Feature Format (GFF) files, and the length of each chromosome was available in the sweet osmanthus genomic database. Then, the 2R-MYB genes were mapped to the corresponding chromosome using MG2C (http://mg2c.iask.in/mg2c_v2.0/). The duplication pattern of each 2R-MYB gene was analyzed using the MCScanX. Ks (synonymous) and Ka (non-synonymous) substitution ratios of gene pairs were assessed using DnaSP v5.0 software [29].

### 2.4. Plant Materials and Transcriptome Sequencing

In this study, four tissue types from sweet osmanthus (‘RiXiangGui’), including roots, stems, leaves (young and mature), and flowers in initial, full, and final flowering stages, were collected. Fresh flowers in the different flowering stages were used to detect the scent compounds with the method of head space solid phase microextraction(HS-SPME) and gas chromatography/mass spectrometer (GC/MS); the detailed information was stated and the results presented in our previous work [15,30]. Altogether, 21 different samples were subjected to Illumina RNA-seq analysis [15]. Reads per kilobase of exon per million reads mapped (RPKM) values were measured to the transcript abundance of each gene.

### 2.5. RNA Extraction and qRT-PCR Analysis

RNA was taken from “RiXiangGui” flowers including those in the bud-pedicel stage (S0), bud-eye stage (S1), initial (S2), full (S3), and final flowering stage (S4) using an RNAprep pure Kit (Tiangen Biotech, Beijing, China) following the manufacturer’s instructions. A sample of 5 μg of the obtained total RNA was used for oligo (dT)18-primed reverse transcription into the first strand cDNA at 42 °C using Revert AidTMM-Mu LV reverse transcriptase (Thermo Scientific, Waltham, MA, USA). The full-length sequence of selected *OfMYB* genes was amplified with Prime STAR (TaKaRa, Dalian, China). 

The first-strand cDNA as mentioned above was diluted 10-fold for qRT-PCR. All primers designed in accordance with each gene’s untranslated region and used for qRT-PCR are listed in Table S1. PCR reactions were performed using an ABI StepOnePlus System (Applied Biosystems, Carlsbad, CA, USA) with the cycles detailed in Xu et al. [31]. *OfRPB2* was used as internal normalization for different flowering stages [31]. The qRT-PCR data were determined with the method described in Yang et al. [32].

Correlation analyses of the expression levels and scent compounds were conducted using SPSS Statistics (Version 22.0; SPSS Inc., Chicago, IL, USA) based on Pearson’s correlation analysis, and *p*-values less than 0.01 were considered extremely significant correlation.

### 2.6. Subcellular Localization of OfMYB Proteins

To observe the subcellular localization of OfMYB proteins, the corresponding open reading frames (ORFs) without the termination codon were amplified. The PCR product was then subcloned into the Super 1300-GFP vector as a C-terminal fusion in-frame with GFP and expressed. The resulting construct was confirmed by sequencing and transformed into *Agrobacterium tumefaciens* strain GV3101. The transformed *A. tumefaciens* lines were infiltrated into six fully expanded leaves of two plants, and every experiment was repeated twice. GFP were observed and imaged using a Zeiss LSM 710 (Carl Zeiss, Jena, Germany) between 36 h and 72 h post-infiltration.

### 2.7. Transcriptional Activation Analysis

In this study, the transcriptional activation of OfMYBs were analyzed using a yeast two-hybrid system. The full-length sequences of OfMYB proteins were cloned and fused in pGBKT7 (Clontech). The empty vector (pGBKT7) was used as a negative control. Then, vectors were transformed into *Saccharomyces cerevisiae* strain AH109 containing the MEL1 reporter, which encoded α-galactosidase. If the recombinant vector pGBKT7-OfMYBs had the transactivation, the expressed MELI reporter gene could be detected. The SD/-Trp medium was used to screen for positive transformants, then the positive clones were further screened on SD/-Trp/-Ade and incubated at 30 °C for 3d. The yeast colonies turned blue on the SD/-Trp/-Ade plates supplemented with X-α-gal (Sigma-Aldrich) when the MEL1 reporter gene was expressed. 

## 3. Results

### 3.1. OfMYB Genes in Sweet Osmanthus

*OfMYB* genes were identified from the sweet osmanthus genome using the MYB domain (PF00249) as a query (http://117.78.20.255/). Approximately 600 protein sequences containing MYB or MYB-like repeats were identified. The Pfam and SMART analyses further confirmed that the domain constituted two MYB repeats. Finally, 243 typical 2R-MYB sequences were obtained after removing sequence without the domain or lacking the N-terminal region. Each gene was checked that it mapped to unique loci in the genome. According to the location of the 243 2R-MYB genes on the corresponding chromosome, the genes were named OfMYB0 through OfMYB242. The basic *OfMYB* gene information, including gene ID, chromosomal location, protein length, MW, pI, and the coding sequences are listed in Tables S2 and S3. The deduced protein length ranged from 89 to 1134 amino acids with MWs of 10,062.45 to 12,4791.4 Da and pI values from 4.74 to 10.54. 

A total of 243 OfMYB amino acid sequences underwent multiple alignment analysis to investigate the features of the domain sequence (Figure S1). The basic region of the 2R-MYB domain of sweet osmanthus contained approximately 108 residues. Regularly distributed and highly conserved tryptophan residues (W) were found in the R2 and R3 repeats. Three tryptophan residues in the R2 region were highly conserved and evenly distributed, whereas the first tryptophan residue in the R3 region was mostly replaced.

### 3.2. OfMYB Gene Location and Duplication Analysis

**Figure 1:**
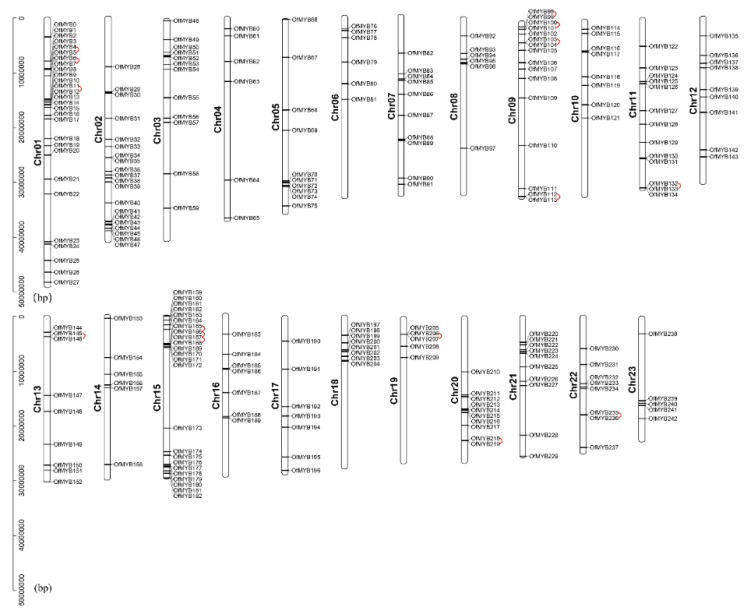
Distribution of 243 2R-MYB genes in sweet osmanthus chromosomes. The physical position of each OfMYB was mapped according to the sweet osmanthus genome. The chromosome number (Chr01–Chr23) is indicated at the left of each chromosome. The red line links the tandem duplication genes. The scale is given on the left.

The physical locations of *OfMYB* genes were localized to the chromosomes (Chr) of sweet osmanthus using MG2C (Figure 1). The *OfMYB* genes were mapped to all 23 chromosomes, but were unevenly distributed. Chr1 contained the largest number of genes (28), followed by Chr15, which contained 24, while only five genes were present on Chr19 and 23. *OfMYB* genes had a relatively high density at the top and bottom regions of Chr15, the top of Chr1, 3, 6, 8, 9, 10, 18, 19, and 21, and the bottom of Chr2, 5, 20 and 23.

**Figure 2:**
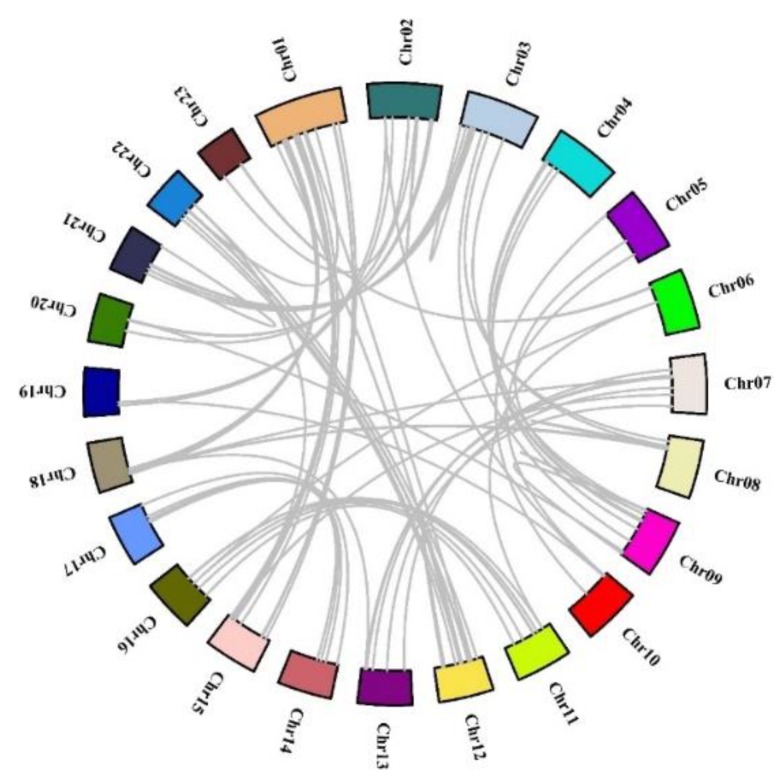
Schematic representations of the interchromosomal relationships of the 2R-MYB genes. Gray lines suggest duplicated MYB gene pairs in the sweet osmanthus genome.

Gene duplication events occurred with plant evolution and thereby derived new gene functions [33]. To understand the collinear relationships of the MYB genes in sweet osmanthus, BLASTP and the MCScanX (Multiple Collinearity Scan) package were used to identify the tandem and segmental duplications (Figure 1 and Figure 2). In total, 15 tandem duplications with 28 2R-MYB genes were found in sweet osmanthus genome. *OfMYB166* and *OfMYB167*, located on Chr12, were shared in three duplications with *OfMYB165* and *OfMYB168*. In combining these data with those from the phylogenetic tree, these tandem gene pairs were found to be located close by on a given chromosome, without intervening annotated genes, and clustered in the same subgroup.

Moreover, seventy-seven gene pairs generated from chromosomal segmental duplications were found and are shown in Figure 2 using Circos [34]. The highest frequency of 2R-MYB gene segmental duplication events occurred between Chr1 and Chr15, including seven segmental duplication events, followed by six between Chr2 and Chr20. Most of the duplicated gene pairs were linked, implying that chromosome or segment duplication might occur among Chr1, 2, 15, and 20.

Ks (synonymous) and Ka (non-synonymous) mutations may occur in the ORF region after gene duplication, resulting in new gene functions. Therefore, the rate of substitution (Ka/Ks) between duplicated gene pairs was calculated to investigate the selection types. The Ka, Ks, and Ka/Ks calculation results for the 77 duplicated pairs are listed in Table S4. The Ks values for the *OfMYB* gene pairs ranged from 0.01 to 1.42. The Ka/Ks values of 2R-MYB paralogous pairs less than one indicated that purifying selection with the segmental duplication was the key driver for the evolution of OfMYB family genes.

### 3.3. The Phylogenetic, Gene Structure, and Motif Analysis of the 2R-MYB Gene Family in Sweet Osmanthus

2R-MYB full-length proteins from *Arabidopsis* (126 members) and sweet osmanthus (243 members) were used to conduct phylogenetic analysis using MEGA 6.0 with the N-J method (Figure S2). Taking into account the classification of AtMYBs [4,5], the *OfMYB* and *AtMYB* genes were divided into 33 subgroups (designated Of1-Of33 in this study), including the previously defined 25 subgroup (S1-S25) categories of *Arabidopsis* and eight new subgroups. As shown in the unrooted tree, the number of 2R-MYBs in the clades ranged from two to 28. Moreover, not all of the sweet osmanthus and *Arabidopsis* 2R-MYB proteins were equally distributed within given subgroups. For example, the subgroups Of6 and Of14 included 17 AtMYB and three OfMYBs. By contrast, eight AtMYBs and 20 OfMYBs were included in subgroup Of33. In addition, species-specific subgroups were observed; Of5 only contained AtMYB members, and those genes were related to glucosinolate biosynthesis in *Arabidopsis*. Of17 contained 10 *OfMYB* genes, in which there were no representatives in *Arabidopsis*.

The OfMYB protein sequences were submitted to MEME, and 20 conserved motifs were identified ranging from 11 to 50 amino acids in length (Figure S3B). The protein sequences of the 20 motifs are presented in Table S5. Almost all the OfMYBs contained motifs 1, 2, 3, and 5; motifs 2 and 3 represent the R2 and R3 MYB domains, respectively.

The analysis of gene structures can help to determine gene functions and confirm phylogenetic relationships within a gene family [35]. Thus, the exon/intron arrangement in the coding region of 243 2R-MYB genes in sweet osmanthus was examined by comparing their genomic and cDNA sequences (Figure S3C). Most of the coding regions were seen to be disrupted by two to 12 introns, while 11 genes contained no introns. Approximately 66% of genes had three exons and two introns. Most of the *OfMYB* genes distributed in the same subgroup possessed similar gene structures. Furthermore, the intron phase, which refers to the splicing position in the MYB domain with regard to codons, was also investigated. Among the 243 MYB domains analyzed, 242 showed phase 0 splicing, 185 showed phase 1 splicing, and 205 showed phase 2 splicing. Moreover, the phases within the same subgroup were mostly conserved during the evolution of *MYB* genes. These data also supported our subfamily designations.

### 3.4. Expression Profiles of OfMYB Genes and Correlation Analysis

**Figure 3:**
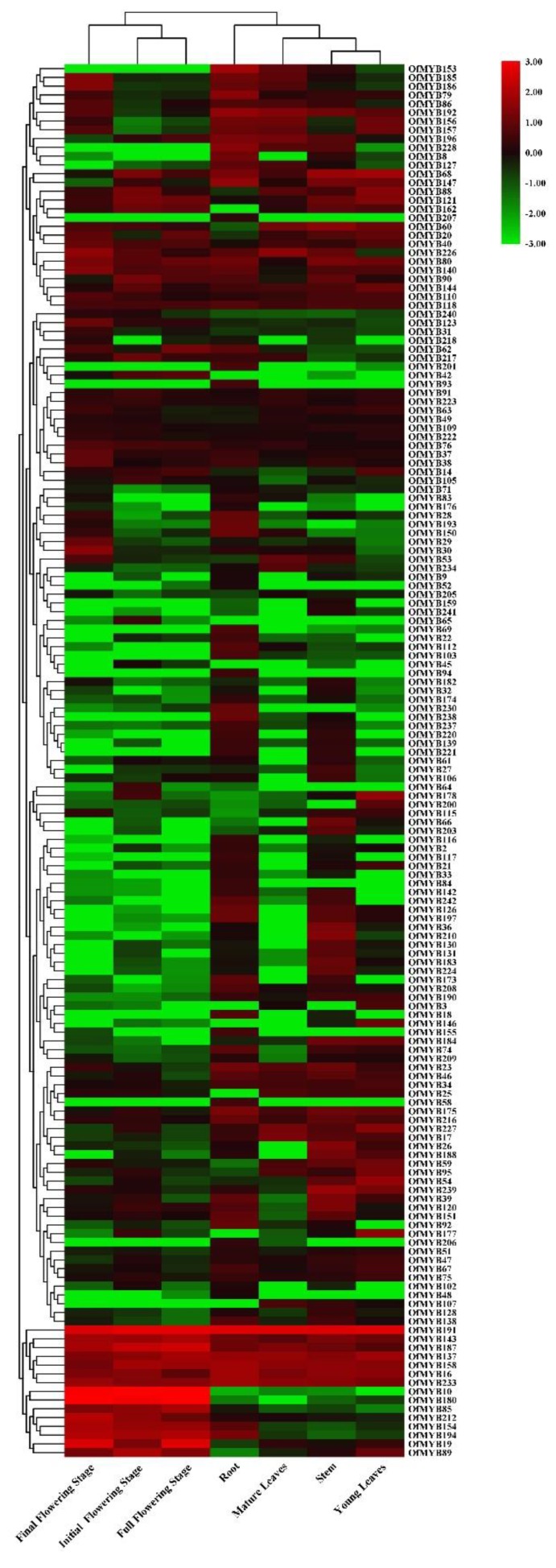
Heatmap of the expression level of sweet osmanthus 2R-MYB genes in tested tissues. The color scale at the right of the dendrogram represents log10 expression values. Red and green colors indicate higher levels and lower levels, respectively.

The expression pattern of a gene provides clues to its function. RNA-Seq data of different tissue types were used to evaluate the transcript accumulation of the 2R-MYB genes. However, the transcripts of 34 genes could not be detected in any samples, which indicated that these may be pseudogenes or expressed under restricted conditions. A total of 49 *OfMYB*s exhibited relatively low transcription levels in one or more tested tissues, suggesting that they may be expressed under restricted conditions. A hierarchical cluster analysis was conducted using the average logarithmic expression values of the remaining 160 OfMYB family members, and the expression profiles were visualized by generating a heatmap (Figure 3). Eleven *OfMYB* genes were constitutively expressed in tested tissues, which suggested that these *OfMYB*s played regulatory roles at different developmental stages (Figure 3; Table S6). A total of 49 *OfMYB*s showed high levels of expression in root, 23 in stem tissues, 18 in leaves, including 16 in young leaf tissue and two in mature leaf tissue. Thirty-five *OfMYB*s were mainly highly expressed in flowers, 12 in the initial flowering stage, 10 in the full flowering stage, and 13 in the final flowering stage.

**Figure 4:**
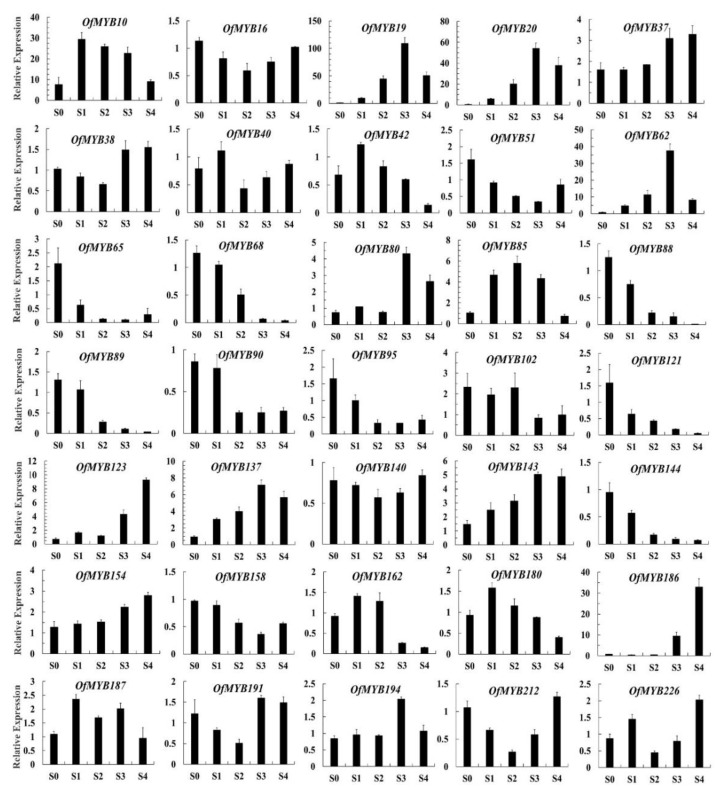
Expression analysis of selected MYB genes using qRT-PCR and gene expression of OfMYBs. S0-S4 correspond to five different flowering stages: bud-pedicel stage (S0), bud-eye stage (S1), initial flowering stage (S2), full flowering stage (S3), and final flowering stage (S4). The x-axis represents the different flowering stages and the y-axis the RPKM values. Data were normalized to the *OfRPB2* expression level. The standard deviations of three biological replicates are represented by the error bars.

**Figure 5:**
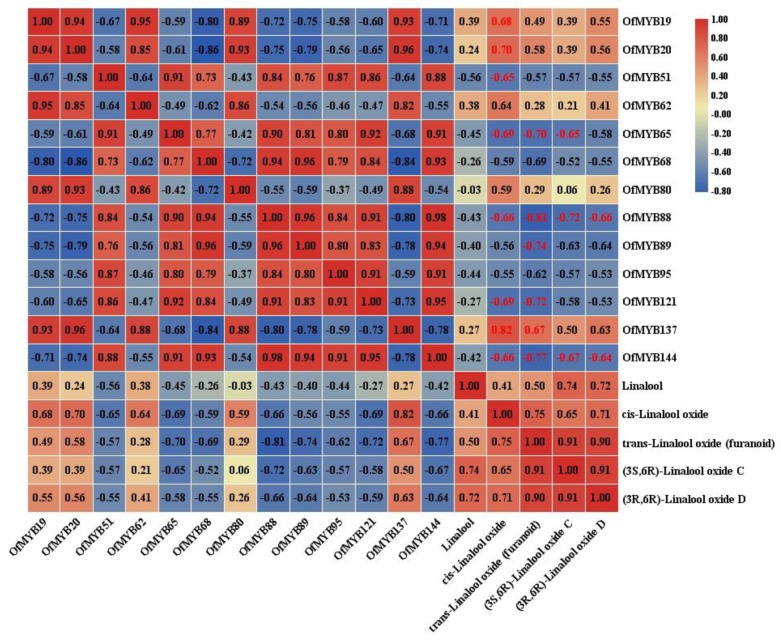
The correlation analysis between the gene expression of OfMYBs and main scent compounds. Red: positively correlated; blue: negatively correlated. Red numbers indicate significant correlations at the 0.01 level.

The 35 *OfMYB* genes with the highest relative expression in flowers were selected for qRT-PCR (Figure 4). The results indicated that the expression levels of *OfMYB10*/*42*/*85*/*180*/*187* showed high transcript levels in bud-eye stage, initial, and full flowering stages, but low transcription in the bud-pedicel and final flowering stages. The expression levels of *OfMYB19*/*20*/*62*/*80*/*137* began to increase as petals opened and peaked in the full flowering stage, then declined in the final stage. This expression pattern concurred with the release of fragrant volatiles. *OfMYB51*/*65*/*68*/*88*/*89*/*95*/*121*/*144* reached their highest levels in the bud-pedicel stage (flower bud), then declined as the petals opened. At the same time, the correlations between the expression patterns of *OfMYB19*/*20*/*51*/*62*/*65*/*68*/*80*/*88*/*89*/*95*/*121*/*137* and the relative contents of linalool and its oxides in different flowering stages, which were determined in our previous research, were studied. *OfMYB19* and *OfMYB20* exhibited remarkably positive correlation with the accumulation of *cis*-linalool oxides, while *OfMYB88* and *OfMYB144* were significantly negatively correlated with the contents of all the linalool oxides. Moreover, *OfMYB51*/*65*/*88*/*121*/*137*/*144* showed significantly negative correlations with one or more linalool oxides (Figure 5).

### 3.5. Characterization of the OfMYB Proteins

**Figure 6:**
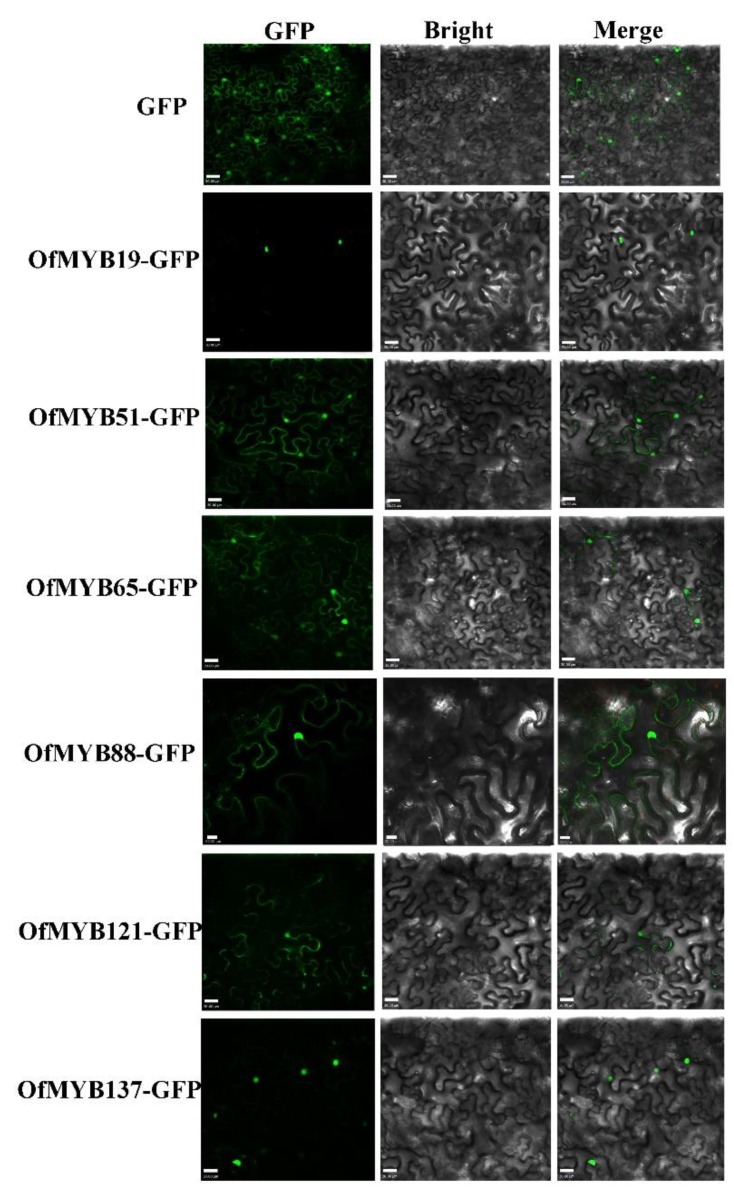
Localization of OfMYB proteins. Subcellular localization analysis of selected 2R-MYB proteins. Transient expression of Super1300-OfMYB fusion and Super1300 construct in tobacco epidermal cells. From right to left, the pictures show fluorescent-field illumination, bright-field, and the overlay of three illuminations. The white block in the lower-left corner is the scale bar.

To explore the subcellular localization of the OfMYB proteins, the full-length sequences of selected genes, *OfMYB19/51*/*65*/*88*/*121*/*137*, were fused to a C-terminal GFP and expressed in the leaf epidermis of *N. benthamiana*; then, GFP signals were observed two days later. As a control, GFP protein was expressed in nuclei and cytosol. As shown in Figure 6, the GFP fluorescence of *OfMYB51*/*65*/*88*/*121* was not only confined in the nucleus, but was also detected on the cell membrane. The green fluorescence produced by OfMYB19 and OfMYB137-GFP was specifically distributed within the nuclei, where it might be involved in regulating transcriptional events. We will conduct further functional investigations of *OfMYB19* and *OfMYB137*. 

**Figure 7:**
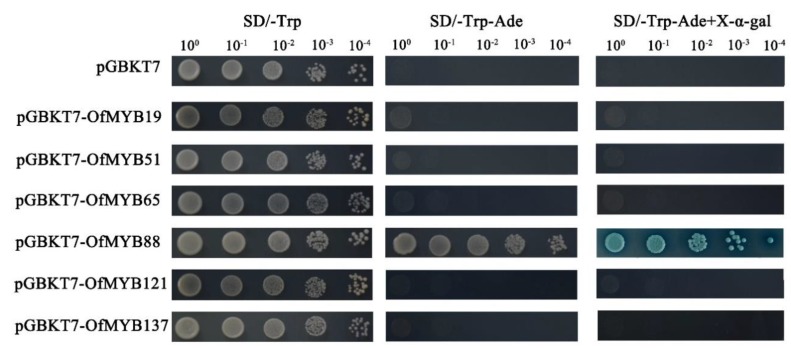
Transcriptional activation activities of OfMYB proteins. The construct of the five vectors pGBKT7-OfMYB was transformed into AH109 and detected on SD/-Trp, SD/-Trp/-Ade, and SD/-Trp/-Ade with X-α-gal.

To identify whether these 2R-MYB proteins had trans-activation activity, the proteins were fused in pGBKT7 to form the BD-OfMYB vector and then transformed into AH109. All yeast cells grew well on the SD/-Trp medium. OfMYB19:pGBKT7/AH109, OfMYB51:pGBKT7/AH109, OfMYB65:pGBKT7/ AH109, OfMYB121:pGBKT7/AH109, and OfMYB137:pGBKT7/AH109 hardly grew on the SD-Trp-Ade plate, which indicated that OfMYB19/51/65/121/137 could have no transactivation in yeast and might need to form a complex with other proteins to perform its transcriptional activation function. OfMYB88:pGBKT7/AH109 grew normally and displayed positive GAL4 activity on X-α-gal-supplemented medium (Figure 7). The results suggested that OfMYB88 exhibited transcriptional activation in yeast.

## 4. Discussion

2R-MYB genes make up the most abundant category of TFs and are involved in various regulatory roles in plants [36]. However, no detailed analysis of 2R-MYB members has been investigated in *O. fragrans*. Here, we conducted a comprehensive study of the 243 2R-MYB genes, including evolutionary relationships, gene structures, motif composition, gene duplication events, chromosome locations, expression profiles, subcellular localization, and transactivation activity.

In general, the MYB protein has a conserved domain at the N-terminus, which is constituted by up to three adjacent repeats, each containing three helices. The second and third helices form the HTH structure combining *cis*-elements [5]. Three tryptophan residues in the R2 repeat were highly conserved and evenly distributed, whereas the first tryptophan residue in the R3 region was mostly replaced (Figure S1). This was consistent with investigations in other species, such as soybean and *Beta vulgaris* [6,37]. In addition, some conserved amino acids were especially distributed in the third helix. Therefore, the conserved third helix could predict that the activity of an MYB gene bound to DNA was stable. The alterations in the third helix could result in targeting genes specifically and/or could affect DNA binding activity.

Phylogenetic analysis showed that OfMYBs and AtMYBs were not equally distributed in the given subgroups. The number of OfMYBs in some subgroups (Of1, Of6, Of9, Of10, Of23, Of26, Of31, Of33) was far greater than that of AtMYBs, which suggested that MYBs were subjected to duplications after the divergence of sweet osmanthus and *Arabidopsis*. This has been observed in pear and soybean [6,7]. The subgroup Of17 did not contain any AtMYB members, which indicated that these MYB family members in sweet osmanthus may have specialized roles. Of5, Of21, and Of27 did not include any OfMYB family members. These homologs were species-specific within a subgroup (Figure 3), which showed ancestral duplication and gene loss events or misannotation of the genome.

Remarkably, genes in the same subgroup generally presented similar intron patterns, including position, distribution, and phases [6]. The first two exons were highly conserved in length, and the third exon, which coded the last region of the R3 repeat and the C-terminal end of the protein, was variable. Changes in length and/or sequences of these exons lead to functional divergence between MYB homologues in plants [38]. The distribution pattern of introns in each subgroup verified the subgroup classification of the phylogenetic tree.

Gene duplications have been deemed crucial to generating new genes in plants and driving genetic evolution [39]. Our results showed that 15 pairs of *OfMYB* genes were identified as tandem duplications and 77 pairs were segmental duplications. The number of OfMYB duplication events suggested that low tandem and high segmental duplication events existed in sweet osmanthus. Segmental duplication events were suggested to be the major causes of *OfMYB* gene expansion. 

The functions of MYB genes from *Arabidopsis* have been annotated extensively. It is possible to predict the functions of OfMYBs by combining expression data with phylogenetic analysis of orthologs from sweet osmanthus and *Arabidopsis*. For instance, the subgroup Of25 consists of three *AtMYB* genes (*AtMYB21*, *AtMYB24*, *AtMYB57*) and two *OfMYB* genes (*OfMYB10*, *OfMYB180*); the AtMYBs in this cluster affect anther development by regulating several pathways [40]. Meanwhile, *AtMYB21* and *AtMYB24* promote petal and gynoecium development [41]. Later, it was reported that these two genes conduced the synthesis of sesquiterpenes [42]. *OfMYB10* and *OfMYB180* showed the highest accumulation of TFs in flower tissue and exhibited differential expression patterns among the three flowering stages. There were two members in Of19 and Of30. Of19 is well known to play an important role in regulating proanthocyanidin biosynthesis in *Arabidopsis* [43]. Therefore, *OfMYB148* might have an effect on the proanthocyanidin biosynthetic pathway of sweet osmanthus. The function of *AtMYB125* is related to pollen formation [44]. Thus, it was expected that Of129 was also involved in pollen formation. In addition, it was observed that 2R-MYB genes in subgroups Of9, Of8, and Of26 in Arabidopsis are involved in biotic and abiotic stress [45,46,47,48].

MYB TFs play vital roles in regulating terpenoids metabolism. For instance, *PtMYB14* is a putative regulator of an isoprenoid-oriented response that contributes to the accumulation of sesquiterpene in conifers [49]. *MsMYB* combines with the promoter of geranyl diphosphate synthase and suppresses its activity to regulate monoterpene production negatively [24]. *AtMYB21* and *AtMYB24* contribute to the production of sesquiterpenes [42]. Overexpression of *VvMYB5b* in tomato induces upregulation of *beta*-carotene [50]. Moreover, several *cis*- elements for MYB binding, especially AC-rich regions bound by 2R-MYBs, were found in the promoter of TPS genes, which is a key enzyme gene for terpenoids. These findings imply that the probable function of OfMYBs was related to terpene biosynthesis in *O. fragrans*. 

In this study, qRT-PCR analysis was conducted to investigate *OfMYBs* involved in terpenoid biosynthesis. *OfMYB* genes (*OfMYB19*/*20*/*137*) presented a similar pattern of expression to the emission of terpenoid volatiles, thus revealing that these genes could play a role in regulating the synthesis of aroma compounds. Notably, *OfMYB51*/*65*/*68*/*88*/*89*/*95*/*121*/*144* reached their highest expression levels in the linggeng stage (flower bud) and declined as the petals opened. The most highly transcribed *OfMYB*s were chosen to perform correlation analysis with linalool and linalool oxides. The expression of *OfMYB19*/*20* coincided with *cis*-linalool oxide production, while *OfMYB51*/*65*/*88*/*121*/*144* demonstrated a significantly negative correlation with the contents of one or more linalool oxides in different flowering stages. Considering these results, *OfMYB19*/*51*/*65*/*88*/*121/137* were analyzed in terms of their subcellular localization and transactivation activity. The results showed that the *OfMYB19* and OfMYB137 proteins were localized in the nuclei and did not show transcriptional activation activity in yeast. Therefore, we hypothesized that OfMYB19 and OfMYB137 might function in a complex with other proteins. Most of the TFs were localized in the nucleus. However, OfMYB51/65/88/121 were dispersed in the nucleus and cell membrane, which was similar to the location results with OsNTL2 [51]. 

## 5. Conclusions

A detailed analysis of 2R-MYB genes was performed in sweet osmanthus. A total of 243 OfMYBs were identified and grouped into 32 clusters with *Arabidopsis* 2R-MYB genes. The reliability of the categories was verified by the distribution of the conserved motif and intron-exons. Most of the subgroups included the *Arabidopsis* and sweet osmanthus MYB proteins, indicating that members of the same subgroup shared a common evolutionary origin and that most MYB genes were functionally conserved during evolution. Meanwhile, gene expansion events in these genomes were indicated, and segmental duplication was suggested to be the major cause of the expansions. An expression profile analysis revealed that *OfMYB* genes were tissue-specific in expression patterns and involved in multiple biological processes. According to the qRT-PCR results and correlation analysis between the expression pattern and the content of linalool and its oxides, the functions of *OfMYB19*/*20*/*137* were potentially related to aromatic volatiles. *OfMYB19* and *OfMYB137* might bind with other transcription factors to exert regulator functions. The systematic analysis provided an overview of the 2R-MYB genes in *O*. *fragrans*. These results will be helpful for understanding and performing further research on the molecular mechanism of OfMYBs related to monoterpenoids biosynthesis, which is of significance for aromatic plant breeding.

## Supplementary Materials

The following are available online at https://www.mdpi.com/2073-4425/11/4/353/s1: Figure S1: Multiple alignments of the 2R-MYB domains in the OfMYB family proteins; Figure S2: Phylogenetic analysis of Arabidopsis and sweet osmanthus 2R-MYB proteins; Figure S3: Phylogenetic tree of *OfMYB* genes, gene structure, and motifs of sweet osmanthus 2R-MYB proteins; Table S1: The primers used in this paper; Table S2: The detailed information of Of2RMYB TFs; Table S3. The coding sequences of the Of2RMYB genes; Table S4: The Ka and Ks values of duplicated OfMYB gene pairs; Table S5: The sequences of 20 conserved motifs in 2R-MYB proteins of sweet Osmanthus; Table S6: Expression profiles of Of2RMYBs in various tissues and three flowering stages; Supplementary File 7: The detailed information about the GC-MS and the fragrant compounds in the different flowering stages of “RiXiangGui”; Supplementary File 8: The detailed information about the transcriptome data.

## Abbreviations

The following abbreviations are used in this manuscript:

TFs	Transcription factors
MYB	v-MYB avian myeloblastosis viral oncogene homolog
(qRT)-PCR	Quantitative real-time PCR
TAIR	The Arabidopsis Information Resource database
SMART	Simple Modular Architecture Research Tool
MW	Molecular weight
pI	Theoretical isoelectric point
ExPASy	Expert Protein Analysis System
GSDS	Gene Structure Display Server
MEME	Multiple Expectation Maximization for Motif Elicitation
GFF	General Feature Format
MG2C	MapGene2Chromosome v2
MCScanX	Multiple Collinearity Scan
Ks	Synonymous
Ka	Non-synonymous
HS-SPME	Head space solid phase microextraction
GC/MS	Gas chromatography/mass spectrometer
RPKM	Reads per kilobase of exon per million reads mapped
